# Association of Ovar‐DRB1 alleles with innate immune responses in sheep

**DOI:** 10.1002/vms3.683

**Published:** 2021-12-09

**Authors:** Atefeh Esmailnejad, Vahid Ganjiani, Elhamsadat Hosseini‐Nasab, Saeed Nazifi

**Affiliations:** ^1^ Department of Pathobiology School of Veterinary Medicine Shiraz University Shiraz Iran; ^2^ Department of Clinical Sciences School of Veterinary Medicine Shiraz University Shiraz Iran

**Keywords:** inflammatory cytokines, innate immunity, MHC, Ovar‐DRB1, sheep

## Abstract

**Background:**

Major histocompatibility complex (MHC) is the best characterised genetic region associated with adaptive immune responses, including humoral and cell‐mediated immunities.

**Objectives:**

In this study, the association of MHC class II alleles with inflammatory cytokines and acute‐phase proteins was evaluated in sheep population.

**Methods:**

Allelic diversity of second exon of ovine DRB1 locus (Ovar‐DRB1.2) was determined in 100 indigenous Iranian Lori‐Bakhtiari fat‐tailed sheep using restriction fragment length polymorphism and direct sequencing methods. The association of DRB1.2 alleles with inflammatory cytokines (interleukin‐1β, IL‐1β; IL‐6 and tumour necrosis factor‐α) and acute‐phase proteins (serum amyloid A, alpha‐1‐acid glycoprotein and haptoglobin) was examined using generalised linear model and multivariate regression analysis.

**Results:**

Seven distinct *RsaI* restriction patterns and fourteen alleles were identified in this population. Allele DRB1*2101 showed a negative influence on the IL‐6 response and was associated with lower serum level of IL‐6. DRB1.2 heterozygous individuals also showed higher haptoglobin concentration than homozygotes.

**Conclusions:**

These results provide additional support for the association between Ovar‐DRB1 alleles and regulation of immune responses in sheep population. Description of MHC polymorphism and its role in the controlling of immune responses will increase our understanding of host–pathogen interactions, and ultimately facilitate the selection of disease‐resistant flocks in genetic breeding programs.

## Graphical abstract

Results of this study provided additional support for the association between Ovar‐DRB1 alleles and regulation of immune responses in sheep population. Description of MHC polymorphism and its role in the controlling of immune responses will increase our understanding of host‐pathogen interactions, and ultimately facilitate the selection of disease‐resistant flocks in genetic breeding programs.

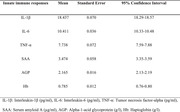

## 1 INTRODUCTION

Major histocompatibility complex (MHC) is a cluster of genes, most of them are responsible for presenting antigens to the immune system and play a central role in regulating the immune responses. MHC class I molecules are cell‐surface receptors that bind intracellular pathogens and present them to the cytotoxic T lymphocytes, generating the cell‐mediated immunity. MHC class II genes encode glycoproteins which bind and present extracellular pathogens to the circulating helper T lymphocytes and initiate humoral immunity (Abbas et al., 2010). Class II loci have been found to be highly polymorphic and considered as a principal target in genotyping studies aimed to evaluate the association of MHC with immune responses and phenotypic traits (Ali et al., 2019; Ashrafi et al., 2014; Cinar et al., 2016; Larruskain et al., 2010; Shen et al., 2014).

MHC in domestic sheep (*Ovis aries*) is known as “Ovar” and physically located on chromosome 20 between bands q15‐q23 (Mahdy et al., 1989). The structure of MHC in sheep is similar to the other mammalian species, including three main classes (I, II and III), each having different functional roles (Amills et al., 1998). Among the Ovar class II genes, DRB1 is highly polymorphic and more than 100 alleles have been identified in the second exon of this locus (Ovar‐DRB1.2) encodes the antigen‐binding cleft of MHC molecules (Ballingall et al., 2008; Ballingall & Tassi, 2010; Gruszczyñska et al., 2005; Konnai et al., 2003). Polymorphism in this exon has offered possibilities for effective immune responses against variety of pathogens (Konnai et al., 2003; Larruskain et al., 2010, 2012; Nagaoka et al., 1999). Research on the MHC as a candidate marker for disease resistance has become a major focus in breeding strategies during the recent years. Numerous investigations have indicated the MHC polymorphism and its involvement in genetic resistance to diseases in different sheep populations (Ali et al., 2019; Herrmann‐Hoesing et al., 2008; Konnai et al., 2003; Larruskain et al., 2012; Li et al., 2010; Stear et al., 2006). Correlation of MHC with some production and reproduction features, such as growth, body weight and fertility has also been documented in sheep (Ashrafi et al., 2014; Cinar et al., 2016; Gruszczyńska et al., 2000).

Determining the role of MHC in controlling the immune responses will provide great information for selection of disease‐resistant population in genetic breeding programs. However, effects of phenotypic traits including immune responses are not consistent across the breeds, and selection based on a specific marker should be applied with great caution. In order to determine the variation in disease resistance or susceptibility among the populations, it is important to characterise the MHC polymorphism and define its relationship to immune responsiveness in each breed. In this study, Ovar‐DRB1.2 genetic diversity was evaluated in an Iranian indigenous sheep breed and the association of DRB1 alleles with innate immune responses, including inflammatory cytokines and acute‐phase proteins, was also investigated in this population. Although correlation of Ovar‐DRB1 alleles with adaptive immune responses against infectious diseases has been demonstrated in sheep population, no evidence of MHC relation to innate immunity has been reported in sheep.

## 2 MATERIALS AND METHODS

### 2.1 Sampling and DNA extraction

One hundred blood samples were collected from six‐month‐old indigenous Iranian fat‐tailed ewes, belonged to the Lori‐Bakhtiari breed, with normal delivery and similar grazing history. Lori‐Bakhtiari breed is originated from Chaharmahal and Bakhtiari province in the west of Iran and kept mainly for meat production. This population was maintained under semi‐extensive farm conditions, with no selection or immigration programs. Vaccination was carried out on the flock as used locally: Brucellosis vaccine (strain Rev.1, Razi, Iran), Anthrax vaccine (strain 34F2, Razi, Iran) and Sheep Pox vaccine (strain RM/65, Razi, Iran) at the age of three months; Ovine and Caprine Clostridial vaccine (polivac®, Vetal, Turkey) at week 8 and repeated 21 days later; Agalactica (Mycoplasma agalactiae, Razi, Iran) at the age of three months and repeated two times with a 3‐week interval.

Blood samples were centrifuged to separate the serum and stored at −20°C until further analyses. Genomic DNA was also extracted from the whole blood using commercial DNA extraction kit (Bioneer, Korea) and purity of the DNA was confirmed by NanoDrop measurement (Thermo Scientific, USA) and agar gel electrophoresis. The study procedure was approved by the Animal Care and Use Committee of the Shiraz University. Flock owner's written consent was obtained for sampling and performing this research.

### 2.2 Ovar‐DRB1.2 genotyping

Second exon of Ovar‐DRB1 locus (Ovar‐DRB1.2) was genotyped using polymerase chain reaction (PCR) restriction fragment length polymorphism (PCR‐RFLP) and direct sequencing. For RFLP analysis, 296 bp fragment of DRB1.2 gene was amplified as described by Konnai et al. (2003). To examine the sequence variability of DRB1.2 locus, PCR products were digested by *Rsa*I restriction enzyme according to the manufacturer's instructions (Fermentas, Germany). The restriction fragments were separated on 15% polyacrylamide gel and *Msp*I‐digested *pBR322* was used as a molecular marker. Fragments were visualised by ultraviolet illumination of SYBR Safe‐stained gels and the fragments’ size were estimated using Photocapture software (version 99.03).

For direct sequencing, PCR amplification was performed using 20 ng of DNA, PCR buffer (20 mM Tris–HCl pH 8.4, 50 mM KCl), 2.5 mM MgCl_2_, 0.25 mM of dNTPs, 1 U of *Taq* DNA polymerase, 20 pmol of forward (DRB1F) (5′‐CGCTCCTGTGAYCAGATCTATCC‐3′) and reverse (DRB1R) (5′‐ACTCACAGTCGTACACACTCG‐3′) primers. The primers are exon spanning, corresponding to the nucleotides 141551–141573 and 142095–142116 of the Ovar‐DRB1 gene (GenBank accession number, EU176819), respectively. After an initial 3 min of denaturation at 94°C, the amplification went through 30 cycles of denaturation at 94°C for 1 min, annealing at 56°C for 1 min and extension at 72°C for 1 min. The amplification was completed with a final extension for 10 min at 72°C. Purification and sequencing of PCR products were performed on an ABI 3730 XL automatic DNA sequencer (Applied Biosystems, Canada). Finally, sequences were aligned using BioEdit software v7.2.0 and compared with other Ovar‐DRB1 sequences from IPD database (http://www.ebi.ac.uk/ipd/mhc/dla/index.html).

### 2.3 Assessment of innate immune responses

Innate immune responses were assessed by measuring the quantitative level of inflammatory cytokines and acute‐phase proteins in the serum samples. Serum levels of interleukin‐1β (IL‐1β), IL‐6, tumour necrosis factor alpha (TNF‐α), serum amyloid A (SAA), alpha‐1‐acid glycoprotein (AGP) and haptoglobin (Hp) were measured by quantitative sandwich enzyme immunoassay method using commercial sheep‐specific kits (Shanghai Crystal Day Biotech, Shanghai, China).

### 2.4 Data analysis

Population genetic analysis including the number of alleles, allele and genotype frequencies, observed and expected homozygosity and heterozygosity for Ovar‐DRB1.2 locus were estimated using Popgene software (Yeh et al., 1997). Deviation of population from Hardy–Weinberg equilibrium (HWE) was also assessed using likelihood ratio test. Unbiased expected heterozygosity and the number of alleles were applied to evaluate the amount of gene diversity in this population (Nei, 1973).

In order to evaluate the possible association and interaction between Ovar‐DRB1.2 alleles and inflammatory factors, generalised linear model (GLM) and multivariate regression analysis were used. Serum level of inflammatory cytokines and acute‐phase proteins were considered as response variables that were specific for each animal. Presence or absence of DRB1.2 alleles in each individual was fitted as a fixed factor and alleles with frequency less than 5% were not included in the model. As immune responses are probably influenced by age and type of birth, these parameters were also fitted as covariates in all of the GLM analyses:

Innateimmuneresponses∼DRB1.2alleles+Age+Typeofbirth

Similarly, two separate GLM analyses included either DRB1.2 genotypes or heterozygosity/homozygosity status as predictors, and innate immune responses as response variables were also performed. The model was calculated for the influence of each predictor separately. To adjust for multiple comparisons, Bonferroni correction was applied and probability less than 0.01 was considered statistically significant. Statistical analyses were performed using SPSS software, version 21 (SPSS Institute, Chicago, IL, USA).

## 3 RESULTS

### 3.1 Ovar‐DRB1.2 genotyping

PCR‐RFLP analysis identified 7 distinct *RsaI* restriction patterns (*a, b, c, d, f, g* and *h*) and 22 genotypes at DRB1.2 locus in Lori‐Bakhtiari population. Pattern *g* had the highest (20.5%) and pattern *h* the lowest (1.0 %) frequency. Genotype *ga* was the most (12%) and *dd* and *ca* the least (1%) frequent genotypes in this population. Ovar‐DRB1.2 sequencing data also revealed 14 alleles in this locus (Table 1). A high level of heterozygosity (82%) and good genotype frequency fit to the HWE was observed in Lori‐Bakhtiari population (*p* = 0.26) (Table 2).

**TABLE 1:** Ovar‐DRB1.2 allele frequency in Lori‐Bakhtiari population

*RsaI* pattern	Frequency (%)	Ovar‐DRB1 allele
*a*	19.5	*0801
*b*	18	*2101
*c*	18.5	*0702,*1202, *1701
*d*	9	*0805
*f*	13.5	*0401, *0402
*g*	20.5	*0301, *0302, *0307, *2001, *2002
*h*	1	*1101

**TABLE 2:** Observed and expected heterozygosity and homozygosity in Lori‐Bakhtiari population

Locus	Sample size	Na	Ne	Obs_Hom	Obs_Het	Exp_Hom^a^	Exp_Het^a^	HWE P‐value	Nei^b^
Ovar‐DRB1.2	100	7	5.777	0.1800	0.8200	0.1689	0.8311	0.2691	0.8269

Abbreviations: Exp_Het, expected heterozygosity; Exp_Hom, expected homozygosity; Na, observed number of alleles; Ne, effective number of alleles; Obs_Het, observed heterozygosity; Obs_Hom, observed homozygosity.

^a^ Expected homozygosity and heterozygosity were computed using Levene (1949).

^b^ Nei's (1973) expected heterozygosity.

### 3.2 Association of Ovar‐DRB1.2 alleles with innate immune responses

Serum level of inflammatory cytokines (IL‐1β, IL‐6 and TNF‐α) and acute‐phase proteins (SAA, AGP and Hb) were measured by quantitative ELISA and presented in Table 3. Using GLM, the effects of Ovar‐DRB1.2 alleles on the innate immune responses were investigated. Pattern *h* (DRB1*1101 allele) was present in 1% of the population and was not fitted in the analyses. GLM analyses revealed a significant influence of DRB1.2 alleles on the innate immune responses in Lori‐Bakhtiari population (*p *< 0.01). Pattern *b* (DRB1*2101 allele) showed a negative effect on the IL‐6 response and was associated with lower IL‐6 serum level (*p* = 0.010). Although pattern *c* showed a positive influence on the IL‐6 titer, it was not statistically significant (*p* = 0.032) (Table 4). No significant association was observed between Ovar‐DRB1.2 alleles and serum amyloid A protein, alpha‐1‐acid glycoprotein, haptoglobin, IL‐1β and TNF‐α responses in Lori‐Bakhtiari population (*p *> 0.01). Analyses of relationship between genetic constitution and immune responsiveness indicated that DRB1.2 heterozygous individuals had higher serum concentration of haptoglobin than homozygotes (β ± SE = 0.69 ± 0.25, *t* = 2.723, *p* = 0.008). No significant differences were observed between DRB1.2 genotypes and innate immune responses in the studied population (*p *> 0.01).

**TABLE 3:** Inflammatory cytokines and acute‐phase proteins in Lori‐Bakhtiari population

Innate immune responses	Mean	Standard error	95% Confidence interval
IL‐1β	18.437	0.070	18.29–18.57
IL‐6	10.411	0.036	10.33–10.48
TNF‐α	7.738	0.072	7.59–7.88
SAA	3.474	0.058	3.35–3.59
AGP	2.165	0.016	2.13–2.19
Hb	0.785	0.012	0.76–0.80

Abbreviations: AGP, alpha‐1‐acid glycoprotein (g/l); Hb, haptoglobin (g/l); IL‐1β, interleukin‐1β (pg/ml); IL‐6, interleukin‐6 (pg/ml); SAA, serum amyloid A (μg/ml); TNF‐α, tumour necrosis factor‐alpha (pg/ml).

**TABLE 4:** Significant association of Ovar‐DRB1.2 alleles with inflammatory cytokines in Lori‐Bakhtiari population

Inflammatory cytokine	Mean ± SD	*RsaI* pattern	DRB1.2 allele	Allele effect	SE	*p*‐Value
IL‐6 (pg/ml)	10.41±0.32	*b*	*2101	−3.451	1.312	0.010
		*c*	*0702,*1202, *1701	2.542	1.167	0.032

Abbreviations: SD, standard deviation; SE, standard error.

## 4 DISCUSSION

Having an effective natural defence system is essential for controlling the potential pathogens and ensuring the animal health (Linde et al., 2008). Exposure to pathogens activates the signalling pathways in innate immune cells and results in the production and secretion of three major inflammatory cytokines, including IL‐1, IL‐6 and TNF‐α. Under the influence of inflammatory cytokines, especially IL‐6, hepatocytes increase the production of acute‐phase proteins such as C‐reactive protein, serum amyloid A, alpha‐1‐acid glycoprotein, haptoglobin, sialic acid and ceruloplasmin. These inflammatory mediators trigger the innate immune responses by recruiting sentinel cells including macrophages and neutrophils to the sites of inflammation, promoting phagocytosis, removal of dead and damaged cells and activating the complement system (Tizard, 2013).

Immune system mechanisms, including innate and adaptive immune responses, are controlled by the influence of multiple genes along with the additive effect of environmental factors. Because of the low heritability and difficulties associated with a reliable evaluation of these traits in animal populations, breeding strategies based on the direct selection of improved immune responses are practically impossible (Boonyanuwat et al., 2006; Notter, 1999). Compared to the traditional selective breeding methods, marker‐assisted selection program attempts to combine the information of genetic markers and quantitative trait loci with the phenotypic data, in order to improve the selection responses. In this program, a trait of interest would be selected based on a genetic marker that is linked to the genes controlling this trait. In other words, genetic markers are used to indicate the presence of a specific phenotypic trait (Ribaut & Ragot, 2007; Wakchaure et al., 2015). Considering the strong correlation between MHC haplotypes and resistance or susceptibility to a wide range of diseases in different animal species, MHC can be considered as a candidate genetic marker for immune responsiveness in breeding strategies.

In this study, the Ovar‐DRB1.2 polymorphism was assessed in indigenous Iranian Lori‐Bakhtiari fat‐tailed ewes using RFLP and direct sequencing methods. Genotyping results indicated a moderate level of polymorphism (seven *RsaI* restriction patterns and 14 alleles) in the studied population. Pattern *h* was only observed in 1% of the studied population. Observed RFLP patterns were in accordance with the previously reported data for several studies concerning the polymorphism of Ovar‐DRB1.2 locus through PCR‐RFLP technique (Gruszczyñska et al., 2005; Konnai et al., 2003; Nikbakht et al., 2012).

Nikbakht et al. (2012) investigated the genetic diversity of Ovar‐DRB1 locus in three Iranian fat‐tailed sheep breeds, including Lori‐Bakhtiari, Shaul and Zandi populations. A total of 7 RFLP patterns (*a*, *b*, *c*, *d*, *f*, *g* and *h*) and 25 alleles, including 7 new alleles (GenBank accession numbers, HQ215209–HQ215215), were identified within 3 populations. Four of seven new alleles were observed only in Lori‐Bakhtiari population and were most similar to DRB*1202, *1701, *1001 and *0102 alleles (Nikbakht et al., 2012). Results of the present study are in close agreement with the reported data, except for the two new alleles (*1001 and *0102) that were not identified in our study. In another study conducted on Iranian Shaul breed, five *RsaI* restriction patterns (*a, b, c, f* and *g*) and eight genotypes were determined which represents the lower polymorphism of this breed compared to the Lori‐Bakhtiari population (Nikbakht Brujeni et al., 2009). Patterns *d* and *h* were not observed in Shaul breed and showed the lowest frequencies (9% and 1%, respectively) in Lori‐Bakhtiari population.

Association of innate immune responses, including inflammatory cytokines (IL‐1β, IL‐6 and TNF‐α) and acute‐phase proteins (SAA, AGP and Hb), with Ovar‐DRB1.2 alleles was also investigated in Lori‐Bakhtiari population. This population was maintained under semi‐extensive farm conditions, with enough exposure to the environmental factors that can stimulate the immune system. Allele DRB1*2101 showed a negative effect on the IL‐6 response and was associated with decreased IL‐6 serum level. DRB1.2 heterozygosity also showed a positive impact on the serum concentration of haptoglobin and supported the heterozygote advantage hypothesis in this population. None of the Ovar‐DRB1.2 alleles showed significant influence neither on the other inflammatory cytokines (IL‐1β and TNFα) nor on the acute‐phase proteins. Lack of such associations in Lori‐Bakhtiari sheep population could propose that the DRB1.2 alleles are not the cause of enhanced or weakened innate immune responses and other genes might have a bigger impact on these traits in sheep. Alternatively, it might have been a consequence of low power inherent in the studies looking at the highly polymorphic genetic markers for complex phenotypic traits. Analysis of the association between genetic markers such as MHC with high level of polymorphism and immune responses can create some problems in the interpretation of results. High level of polymorphism requires large number of comparisons that could lead to the loss of power and low probabilities might arise by chance (Stear et al., 2006). However, analysing the limited number of RFLP patterns (NO = 6) instead of high number of DRB1.2 alleles (NO = 14) reduced the problem of multiple comparisons to some extent. Moreover, Bonferroni correction was included in the statistical analyses and probability less than 0.01 was considered statistically significant as the practical solutions to address the problem of multiple comparisons. Finally, immune responses could be influenced by different factors including sex, age, type of birth, nutritional status, environmental conditions, sire, dam and other background genetic effects. In order to have a more accurate association result, these parameters should be also included in the analysis.

Zamani et al. (2016) studied the association of Ovar‐DRB1.2 alleles with some production traits and immunological parameters in Iranian Mehraban and Lori‐Bakhtiari sheep populations. DRB1 genotypes showed a significant correlation with serum total protein, total globulins, α2‐globulin, β‐globulin and γ‐globulin. The findings of this study indicated that the mutation in DRB1.2 region had a considerable impact on immunity, and to a less extent, on production traits (Zamani et al., 2016). Most relevant studies on Ovar polymorphism and disease resistance and/or susceptibility have mainly focussed on DRB1 locus and significant associations have been reported, the majority being concerned with gastrointestinal nematodes and cestodes (Ali et al., 2019; Shen et al., 2014; Valilou et al., 2015). Allele DRB1*1101 has been associated with drastic reduction in faecal egg counts following infection with *Teladorsagia circumcincta* (Ali et al., 2019). A strong correlation between DRB1 alleles and hydatidosis (*cystic echinococcosis*) resistance and susceptibility has also been reported in different sheep populations (Li et al., 2010; Shen et al., 2014). Likewise, there are a few reports on the involvement of Ovar‐DRB1 alleles in resistance to bacterial and viral diseases including footrot (Escayg et al., 1997; Valilou et al., 2016), Johne's disease (Reddacliff et al., 2005), ovine progressive pneumonia (Herrmann‐Hoesing et al., 2008), bovine leukaemia (Konnai et al., 2003), Maedi‐Visna (Larruskain et al., 2010) and pulmonary adenocarcinoma (Larruskain et al., 2012). However, this study is the first research attempted to link the Ovar‐DRB1 alleles to the innate immune responses in sheep.

## 5 CONCLUSION

Use of genetic markers such as MHC for selecting improved immune responses has shown the promising results in the breeding programs. In the present study, moderate genetic diversity and high level of heterozygosity were observed at the DRB1 locus in indigenous Lori‐Bakhtiari population. Allele DRB1*2101 was negatively associated with the IL‐6 response in the studied population. However, further investigations are necessary to determine whether this allele contains the causative mutations or is only a marker in linkage disequilibrium with a causative variant. Understanding the indigenous breeds’ genetic patterns of MHC and their associations with immune responses seems to be worthwhile with respect to the conservation and genetic improvement of sheep populations.

## AUTHOR CONTRIBUTIONS

**Atefeh Esmailnejad**: Conceptualisation; data curation; formal analysis; funding acquisition; investigation; methodology; project administration; resources; software; supervision; validation; visualisation; writing – original draft; writing – review and editing. **Vahid Ganjiani**: Formal analysis; investigation; methodology; resources; visualisation; writing – original draft. **Saeed Nazifi**: Conceptualisation; data curation; formal analysis; funding acquisition; investigation; methodology; project administration; resources; software; supervision; validation; visualisation.

## ETHICAL APPROVAL

The Animal Care and Use Committee of the Shiraz University approved the experimental procedures which are in compliance with the regulations for protection of animal research.

## CONFLICT OF INTEREST

The authors declare that there is no conflict of interest.

### PEER REVIEW

The peer review history for this article is available at https://publons.com/publon/10.1002/vms3.683

## Data Availability

The data that support the findings of this study are available on request from the corresponding author.
